# Retrieval of Crop Variables from Proximal Multispectral UAV Image Data Using PROSAIL in Maize Canopy

**DOI:** 10.3390/rs14051247

**Published:** 2022-03-03

**Authors:** Erekle Chakhvashvili, Bastian Siegmann, Onno Muller, Jochem Verrelst, Juliane Bendig, Thorsten Kraska, Uwe Rascher

**Affiliations:** 1 Institute of Bio- and Geosciences: Plant Sciences (IBG-2), Forschungszentrum Jülich GmbH, 52428 Jülich, Germany; 2 Image Processing Laboratory (IPL), University of Valencia, Paterna, 46980 Valencia, Spain; 3 Institute for Crop Science and Resource Conservation, University of Bonn, Campus Klein-Altendorf 1, 53359 Rheinbach, Germany

**Keywords:** UAV, multispectral, radiative transfer model, inversion, PROSAIL, leaf area index, leaf chlorophyll content, canopy chlorophyll content

## Abstract

Mapping crop variables at different growth stages is crucial to inform farmers and plant breeders about the crop status. For mapping purposes, inversion of canopy radiative transfer models (RTMs) is a viable alternative to parametric and non-parametric regression models, which often lack transferability in time and space. Due to the physical nature of RTMs, inversion outputs can be delivered in sound physical units that reflect the underlying processes in the canopy. In this study, we explored the capabilities of the coupled leaf–canopy RTM PROSAIL applied to high-spatial-resolution (0.015 m) multispectral unmanned aerial vehicle (UAV) data to retrieve the leaf chlorophyll content (LCC), leaf area index (LAI) and canopy chlorophyll content (CCC) of sweet and silage maize throughout one growing season. Two different retrieval methods were tested: (i) applying the RTM inversion scheme to mean reflectance data derived from single breeding plots (mean reflectance approach) and (ii) applying the same inversion scheme to an orthomosaic to separately retrieve the target variables for each pixel of the breeding plots (pixel-based approach). For LCC retrieval, soil and shaded pixels were removed by applying simple vegetation index thresholding. Retrieval of LCC from UAV data yielded promising results compared to ground measurements (sweet maize RMSE = 4.92 μg/cm^2^, silage maize RMSE = 3.74 μg/cm^2^) when using the mean reflectance approach. LAI retrieval was more challenging due to the blending of sunlit and shaded pixels present in the UAV data, but worked well at the early developmental stages (sweet maize RMSE = 0.70m^2^/m^2^, silage RMSE = 0.61m^2^/m^2^ across all dates). CCC retrieval significantly benefited from the pixel-based approach compared to the mean reflectance approach (RMSEs decreased from 45.6 to 33.1 μg/m^2^). We argue that high-resolution UAV imagery is well suited for LCC retrieval, as shadows and background soil can be precisely removed, leaving only green plant pixels for the analysis. As for retrieving LAI, it proved to be challenging for two distinct varieties of maize that were characterized by contrasting canopy geometry.

## 1 Introduction

With the recent technological advancement of UAV platforms and diversification of sensor types, it became possible to retrieve and map crop biophysical variables from high-resolution imagery (<0.1 m) [1]. Biophysical and biochemical variable maps can support agricultural field management, by providing vital information on the plant status throughout the growing season [2]. Apart from supporting farmers in decision-making, spatial information on crop biophysical variables derived from UAV image data can assist the plant breeding community in the high-throughput phenotyping of large quantities of breeding plots [3,4]. Leaf area index (LAI), which is linked to the absorption of photosynthetically active radiation, transpiration, energy exchange and other phytophysiological variables [5,6], is one of the most widely explored structural state variables in the crop modeling community. Similar to LAI, leaf chlorophyll content (LCC) can also deliver crucial information on photosynthetic capacity, primary production and the nitrogen status [7–9]. For this reason, quantification of both variables is important to monitor the crop’s status.

Various plant variable retrieval methods have been developed in the remote sensing community in the past few decades [10]. From these methods, parametric regression models have been used to establish an empirical relationship between the variable of interest and vegetation indices, such as for retrieving LAI [11,12] and LCC [2]. Nonparametric models, e.g., machine learning algorithms, have also been explored to retrieve biophysical and -chemical variables [13–15]. Often, these methods are characterized by a lack of transferability in space and time and to different environmental conditions and sensors, which might limit their usability to the datasets they were calibrated for. Unlike these methods, vegetation radiative transfer models (RTMs), which describe the radiation interaction with the canopy based on physical laws, are not bound by the constraints of geographical location, time of data acquisition or the sensor configuration. Compared to parametric regression models, RTMs could make use of the whole spectrum, instead of exploiting subsets of the electromagnetic spectrum. This ability is crucial since plant biophysical variables are sensitive across spectral domains, not at specific wavelengths [16]. The inversion of RTMs allows us to derive a large variety of state variables from multi- and hyperspectral remote sensing image data. RTMs have been successfully applied to airborne and satellite data to retrieve plant variables [17,18].

Multiple vegetation RTMs with varying complexity have been developed for specific purposes and are thus bound by conceptual and computational assumptions [19]. For example, 3D radiative transfer models enable us to calculate the radiation budget in a more complex 3D plant canopy, considering the vertical profile and shadows, but require a higher number of input variables to be taken into account [20]. Due to this fact and the demand for more computing power, until now, they have been mainly used for vegetation variable retrieval in complex canopies within a limited spatial extent [21,22]. In contrast, the combination of the leaf RTM PROSPECT-D [23] and the canopy bidirectional reflectance model (4SAIL) [24], known as PROSAIL [25], requires only a few input variables. However, the assumptions imposed by the simplicity of the model limit its applicability to various scenarios and complex canopy types. Nevertheless, PROSAIL offers a well-balanced compromise between model complexity and required computational effort, and therefore is especially efficient when it comes to large images [26].

In order to retrieve plant variables, the RTM inversion scheme needs to be applied to the reflectance data. RTM inversion is inherently an ill-posed problem: similar simulated reflectance spectra can lead to a wide range of solutions [27]. To overcome the issue of illposedness, several inversion schemes have been proposed: numerical optimization (i), look-up table (LUT)-based inversion (ii) and hybrid approaches (iii). (i) Numerical optimization minimizes a cost function value between the measured and predicted reflectance spectrum in an iterative manner. This method requires significant computing power and is time-intensive if applied to a huge number of pixels. (ii) In contrast, the LUT-approach uses a high number of simulations to produce several hundred or thousand reflectance spectra from numerous combinations of input variables. The subsequent inversion is based on finding the best match between a simulated and measured reflectance spectrum by applying a cost function, with the aim to minimize the summed error between a simulated and observed reflectance spectrum. The approach requires a moderate amount of time to build up the LUT, but the subsequent error minimization is very fast [28]. (iii) Hybrid approaches combine the fast computation power of machine learning and generalization level of RTMs [10]. In this approach, RTM simulations are used as training data, leaving ground measurements only for validation.

Ill-posedness can be further alleviated by constraining the variables during the LUT generation. Variables can be constrained based on a priori knowledge about a certain variable and its value range observed in the field [27,29]. Another way to reduce ill-posedness is exploring different cost functions [10,30], the application of multiple best solutions instead of a single best solution [31,32] and adding artificial noise to include uncertainties during the measurements [30,33].

The applicability of PROSAIL to UAV data has been explored in various studies for various crops. The most widely retrieved variables are green fraction (GF), LAI, LCC and canopy chlorophyll content (CCC) [34–39]. In the majority of these studies, UAVs were either flown at high altitudes to produce coarse-resolution imagery that mimics airborne or satellite data [34,35,38], or the resolution of the final orthomosaic was artificially reduced [34]. This reduction was done to meet the assumption of a turbid medium model such as PROSAIL. While reducing the spatial resolution might be sensible for structural variable retrieval, it has an adverse effect on the estimation of biochemical variables: image data become affected by mixed pixels, meaning that it is no longer possible to separate shaded and soil pixels from vegetation (leaves). This blending effect in turn influences the proper estimation of leaf variables per plot. Images of higher spatial resolution allow the separation of only vegetated pixels from the scene to better estimate the leaf variables. Additionally, in the majority of the above-mentioned studies, hyperspectral sensors were employed. While these sensors deliver spectrally contiguous data, their applicability in breeding and precision agriculture is currently limited due to their high cost and complex data post-processing. Multispectral sensors, on the other hand, are much cheaper and provide information of important spectral regions, which proved to be sufficient to retrieve crop biophysical variables of comparable quality [35]. A common method of retrieving vegetation variables at plot level is averaging the measured spectra per plot and applying the inversion scheme to it. While this method works well for coarse-resolution imagery and for structural variables, we assume that leaf biochemical variables could be better estimated by applying the inversion scheme to the reflectance maps and then averaging the variables per plot. In this way, valuable information on each canopy feature can be obtained.

Therefore, the objective of this study is to explore the potential of PROSAIL LUT inversion for estimating LAI, LCC and CCC from spatial high-resolution multispectral imagery. The specific questions that we aim to answer are:

1. How well can LAI, LCC and CCC be retrieved from high-resolution UAV multispectral image data for complex canopies such as maize?
2. Which inversion scheme, mean reflectance (applying the scheme to a single spectrum averaged per plot) or pixel-based approach (applying the scheme to every pixel and then averaging), leads to more accurate results?
3. How does the retrieval accuracy vary within the growing season for different growth stages?

For this purpose, we have acquired a time-series of image data over a maize canopy throughout the growing season in 2020. Two types of maize (sweet and silage), which have contrasting structural and functional canopy traits, were sampled during the vegetation period. We have compared estimated variables to non-destructive ground measurements of LAI and LCC conducted shortly before or after UAV data acquisitions.

## 2 Materials and Methods

### 2.1 Study Area

**Figure 1:**
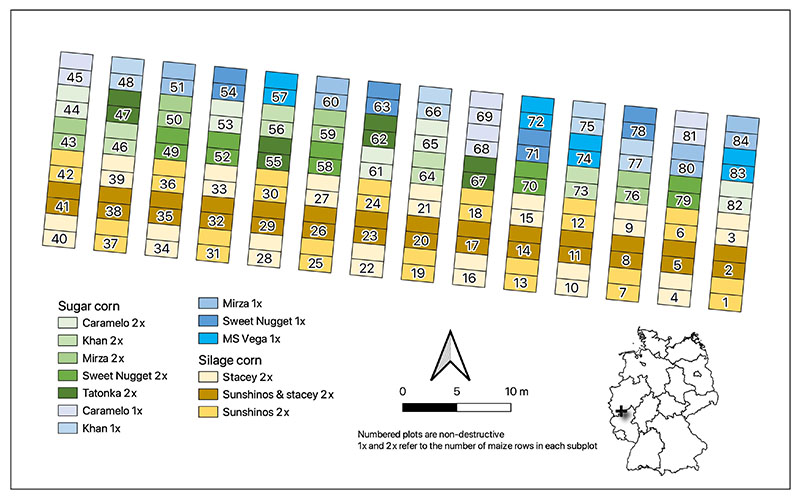
Map of the maize trial in PhenoRob Central Experiment, at agricultural research station of campus Klein-Altendorf. Two-row sweet maize plots are depicted in green, one-row sweet maize plots are depicted in blue, and silage maize plots are shown in yellow gradient colors. Inset map shows the location of the experimental field within Germany.

The study area is located at the agricultural research station Campus Klein-Altendorf in the western part of Germany (50°37′ N, 6°59′E, altitude 176 m) (Figure 1). The average annual precipitation is 603 mm and the long-term average annual temperature is 9.4 °C. Two cultivars of silage (*Zea mays*) and seven cultivars of sweet maize (*Zea mays* convar. *saccharata* var. *rugosa*) were sown in a field experiment. The total area of the maize trial was 0.12 ha. Silage maize was represented by two varieties, Sunshinos and Stacey, and a mixture of these varieties. Sweet maize was represented by seven varieties: Caramelo, Khan, Mirza, Sweet Nugget, Tatonka, Sweet Nugget and MS Vega. A total of 84 plots, each with the size of 3 × 3 m, were divided into non-destructive and destructive subplots, each 1.5 × 3 m in size. Moreover, 23% of the total plots had two maize rows. The varieties Sweet Nugget and MS Vega were sown in 1 row per subplot. The other sweet maize varieties, except for Tatonka, were sown either in two or one rows. The middle plots of the silage maize part of the trial were a mixture of two silage maize varieties.

### 2.2 Aerial Campaigns

A DJI Matrice Pro 600 (SZ DJI Technology Co., Ltd., Shenzhen, China) served as a sensor carrier platform. The MicaSense Dual camera system, consisting of two multichannel cameras—the MicaSense RedEdge-MX and the RedEdge-MX Blue (AgEagle Sensor Systems Inc.,Wichita, KS, USA)—was mounted on a Ronin MX gimbal attached to the UAV. The cameras capture images synchronously in ten spectral bands and store them as separate image files (Table 1). Each camera has a field of view (FOV) of 47.2° and focal length of 5.4 mm. The images were geotagged with the help of a global navigation satellite system (GNSS) receiver mounted on the UAV. A downwelling light sensor (DLS) provided by the camera manufacturer was installed at the top of the UAV. The DLS measures irradiance for each band and saves this information in the image metadata. The UAV acquired image data at 20 m above ground level, which resulted in 1.39 cm ground sampling distance (GSD). The flight altitude was set as low as possible to ensure high spatial resolution, but high enough to guarantee proper scene reconstruction with a sufficient number of matching features in image pairs. The UAV was flown at a speed of 3 m/s, resulting in a forward overlap of 80% and sidelap of 70%. Setting these parameters to be high is crucial for achieving good geometry and successful scene reconstruction. Flights were conducted around solar noon on days with stable illumination conditions (Table 2). A set of nine near-Lambertian panels (Mankiewicz Gebr. & Co. (GmbH & Co. KG), Hamburg, Germany) with varying reflectance factors and a flat spectral response across the VNIR spectral range, ranging from dark (2%) to bright (63%), was placed within the experiment on bare soil before each flight. The panels were recorded from the same height as the experimental plots (20 m). During post-processing, the panels were used to convert the radiance orthomosaic of the study area to top-of-canopy reflectance [40]. Panel reflectances were measured in the field on 23 June 2020 under sunny conditions using an ASD FieldSpec 4 spectroradiometer (Malvern Panalytical, Malvern, UK). The collected spectral measurements were resampled to match the spectral bandwidths of the MicaSense sensor. In total, eight flight campaigns were carried out throughout the growth season of maize, which lasted from early June until the harvest in late September (Table 2). The measurement dates covered principal growth stages of maize (BBCH scale), which are important from an agronomic perspective for fertilizer application, irrigation and disease control: leaf development, stem elongation, inflorescence, flowering fruit development and ripening.

To assess the illumination conditions during the flights, we mapped the irradiance measurements collected by the DLS. A seamline file was exported from Agisoft MetaShape (v. 1.6.5 photogrammetric software, Agisoft LLC, St. Petersburg, Russia), containing the information for the image footprint that was used to create an orthomosaic. Seamlines corresponded to the borders between parts of images that were used for orthophoto generation. The mosaic blending mode was chosen to minimize optical disruption, i.e., to generate a smooth orthomosaic without any texture differences between blended images. The DLS measurements were assigned to each respective footprint. Due to rapidly changing illumination conditions, the dataset recorded on 14 July was removed from further processing. Please refer to the irradiance map of 14 July in Appendix A, Figure A2.

### 2.3 Image Processing

Raw MicaSense images were converted to radiance using the equation provided by the camera manufacturer [41],

(1) $$L=V\left(x,y\right)*\frac{a_{1}}{g}*\frac{p-p_{BL}}{t_{e}+a_{2}y-a_{3}t_{e}y}$$

where *L* is the radiance in W/m^2^/sr/nm, *V(x, y)* is the vignette polynomial function for pixel location *x, y*), *a*_1_, *a*_2_, *a*_3_ are the radiometric calibration coefficients, *g* is the sensor gain setting, *p* is the normalized raw pixel value (divided by 2N, where N is the number of bits in the image), *x* and *y* are the pixel row and column numbers, respectively, *p_BL_* is the normalized black level value, and *t_e_* is the image exposure time in ms. The MicaSense python library [42] was used to convert raw images to radiance.

Radiance images were stitched in Agisoft Metashape. They were georectified using 15 ground control points (GCPs) distributed in the field. GCPs were measured with a real-time kinematic GNSS (Trimble R4 GNSS system) with a horizontal accuracy of 8 mm and vertical accuracy of 15 mm. Orthomosaics were generated with mosaic blending mode enabled and exported with the highest resolution shared between all flight campaigns (0.015 m). To correct spectral data for atmospheric attenuation with the empirical line method (ELM) [43], mean radiance values were extracted from the central parts of the panels located within the orthomosaic. Panels with a reflectance factor of >22% were saturated in the visible spectral bands and thus were not considered in the ELM. The same bright panels were not saturated in red-edge and NIR regions and were used in ELM. A simple linear regression was calculated based on the mean radiance values of the panels in the orthomosaic and the measured and resampled reflectance spectra of the panels collected with the ASD spectroradiometer. The resulting linear equations determined for each spectral band were applied to the orthomosaic to convert the radiance values of the image pixels to reflectance, which were used as the basis for the RTM inversion.

For the retrieval of leaf chlorophyll content, shaded and soil pixels were removed from the orthomosaics using a threshold of 0 based on the Hue index [44] in the R package FIELDimageR [45]. Soil removal was problematic for the dataset acquired on 14 September, since a considerable number of leaves in sweet maize were brown and had a spectral reflectance similar to soil. Thus, soil removal with the index thresholding approach also removed brown leaves from the scene. To avoid this, a crop surface model approach [46] was explored. We subtracted the digital terrain model (DTM) produced from the image dataset acquired before sowing from the digital surface model (DSM) based on the dataset recorded on 14 September to create a crop height model. We removed all pixels with values close to zero from the scene by employing manual thresholding and applied the resulting mask to the orthomosaic.

### 2.4 Field Measurements

Field measurements were conducted before and/or after each aerial campaign. On every measurement date, 18 plots were sampled (seven silage and eleven sweet maize plots). From 30 July onward, more plots (Table 3) were measured. LAI was sampled non-destructively using the SunScan plant canopy analyzer (Delta-T devices Ltd., Burwell, United Kingdom). The SunScan system consists of a probe, a sunshine sensor and a personal digital assistant (PDA). The probe has 64 PAR sensors placed on a 1-m-long probe. During data acquisition, the measurements of all sensors are transferred to the PDA. A sunshine sensor, transmitting information on direct and diffuse light to the probe via radiolink [47], was placed between the plots at a height of 1.5mto avoid shading. The probe was used to collect measurements at six different positions in a non-destructive subplot. According to the SunScan user instructions for each reading, the probe was diagonally placed on the crop rows [47]. The ellipsoidal leaf angle distribution parameter (ELADP) value was set to 1.37, based on the average value for maize taken from the SunScan user manual [48]. Measurements were performed in the period three hours before to three hours after solar noon, as recommended by the manufacturer. LAI readings were averaged for each subplot.

Chlorophyll measurements were collected with a SPAD-502 Chlorophyll Meter (Konica Minolta, Tokyo, Japan). In each subplot, five plants were randomly selected to measure the five upper leaves. Each leaf was measured three times by placing the SPAD at the base, middle and the tip of the leaf. All SPAD readings were averaged per plot. The calibration equation that was used to convert SPAD readings into chlorophyll content (μg/cm^2^) has been adopted from Haboudane [2]:

(2) $$LCC=9.1411e^{0.0318*SPAD}$$

CCC is the product of LAI and LCC:

(3) CCC=LCC∗LAI

### 2.5 LUT-Based PROSAIL Inversion

Variable retrieval was conducted in the ARTMO toolbox (Automated Radiative Transfer Models Operator) [49]. The ARTMO toolbox is a software package written in MatLab that provides various tools for running different RTMs in forward or inverse mode, both at leaf and canopy scale. We used PROSAIL [25], a coupled RTM model consisting of the leaf model PROSPECT-5 [23] and the canopy model 4SAIL [50].

For the retrieval of LAI, LCC and CCC, PROSAIL model inversion was conducted using a LUT-based approach. To parameterize the model, we used the variable ranges observed in the field during each measurement date (Table 3) or took values from the literature. Separate LUTs were constructed for each date to constrain the model and reduce the effect of ill-posedness [27].

Leaf structure index N was set to a value range of 1.2–1.8, as has been reviewed in the literature for maize [26]. Brown pigment content was set to zero for the datasets acquired at early crop development. For datasets from 19 August onward, the range of 0–0.5 with uniform distribution was used, as the leaf browning was observed in the majority of the sweet maize plots. The range of dry matter content was set to 0.004–0.0075 (g/cm^2^), according to a literature review [26]. Since silage and sweet maize had visually different leaf inclination angles, the leaf angle variable was set to a range of 20–70. Silage maize varieties had predominantly erectophile/spherical leaves, while sweet maize leaves had predominantly planophile/spherical leaves. Soil reflectance information was extracted from pure soil pixels from the radiometrically calibrated orthomosaics. From every orthomosaic, several areas with visually differentiable soil characteristics (compressed, ploughed) were extracted using the mean reflectance of different regions of interest. Soil spectra were extracted for dry and wet soil separately. For LCC retrieval, the soil reflectance was of low importance in this study, as the high-resolution image data allowed us to easily differentiate soil from plant pixels and remove them when necessary. The hot spot parameter was set to the range given in the literature of 0–0.2 [26]. Fixed sun zenith angles were used as input for different dates. Observer zenith angle (OZA) was fixed to 0°, as the images were acquired from the nadir position. We did not calculate the OZA [34] for every pixel as it was beyond the scope of this study. However, the reader should keep in mind that in the generation of orthomosaics, not only the nadir part of the images was used but, to some degree, the peripheral parts as well. To produce LUTs, PROSAIL was first run in forward mode separately for each date. We used either Gaussian or uniform distributions for the variables (Table 4). Multiple studies have successfully used Gaussian distributions for chlorophyll content retrieval [51]. It has been demonstrated that applying sampling constraints to the LUT generation based on a priori information may increase the retrieval accuracy [27]. Combination of these variables resulted in LUTs having hundreds of thousands of entries. We used the Latin hypercube sampling (LHS) [52] method implemented in ARTMO to select only a subset of 10,000 entries for each LUT.

**Figure 2:**
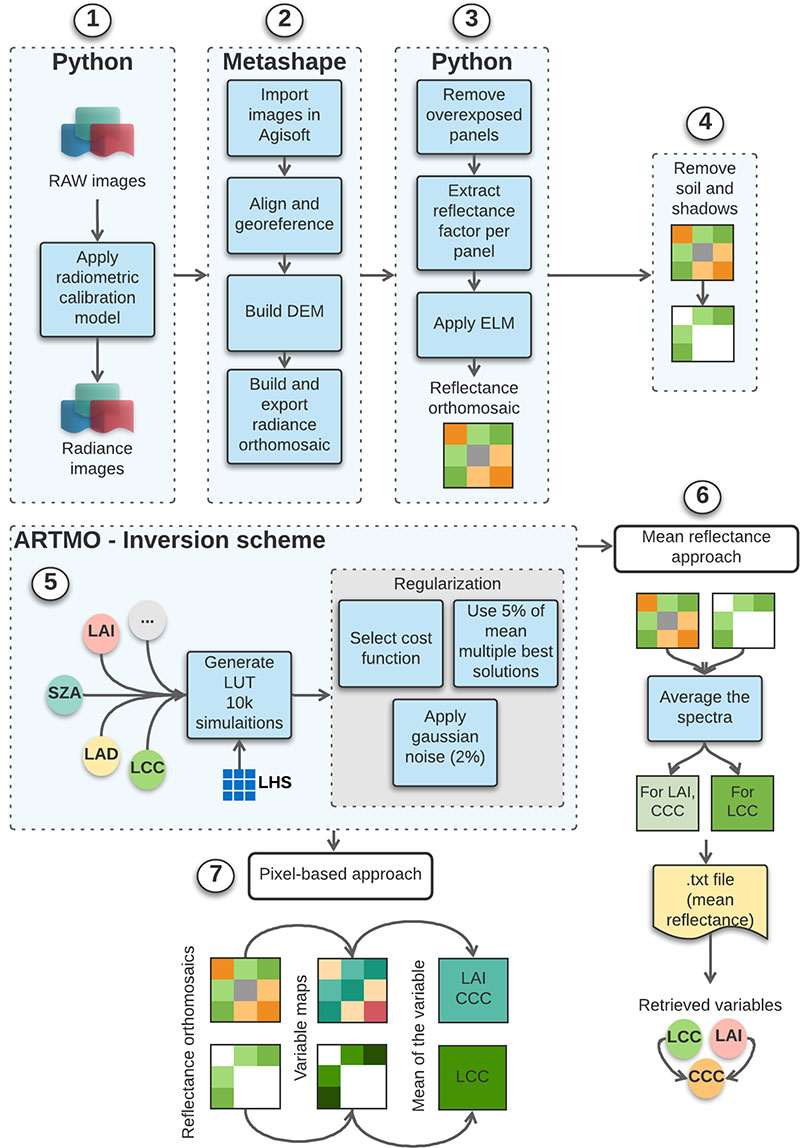
General workflow for the retrieval of LAI and chlorophyll content using different software packages: (1) conversion of raw images to radiance, (2) scene reconstruction in photogrammetric software, (3) application of ELM, (4) soil/shadow removal for pigment retrieval and (5) LUT construction and inversion, (6–7) application of inversion scheme using two different approaches. LHS—Latin hypercube sampling.

Gaussian noise of 2% was added to the LUTs to account for variable measurement uncertainties. We used the mean (5%) of the multiple best solutions to find the best match between simulations and measurements to reduce the effect of ill-posedness. Two different approaches were applied to retrieve the variables: (1) the inversion scheme was applied to mean reflectance spectra calculated for each plot (mean reflectance approach) to retrieve the variables; (2) the inversion scheme was applied to orthomsoaics with reduced spatial resolution (GSD = 0.09 m) to retrieve LAI and CCC, with the aim to speed up the mapping procedure. It was also applied to the orthomosaics with the original spatial resolution to map LCC. Afterwards, mean LCC values for each plot were determined and compared to the ground measurements (Figure 2).

### 2.6 Statistical Analysis

To study the accuracy of the plant trait retrieval, several metrics were employed: root-mean-square error (RMSE), relative RMSE (rRMSE) and the coefficient of determination (r^2^):

(4) $$RMSE=\sqrt{\frac{1}{n}\sum_{i=1}^{n}{\left(d_{i}-f_{i}\right)}^{2}}$$

(5) $$rRMSE=100*\frac{RMSE}{\langle d\rangle }$$

where *d_i_* are observations, *f_i_* are the estimates and 〈*d*〉 is the statistical mean.

## 3 Results

### 3.1 Variable Retrieval

**Figure 3:**
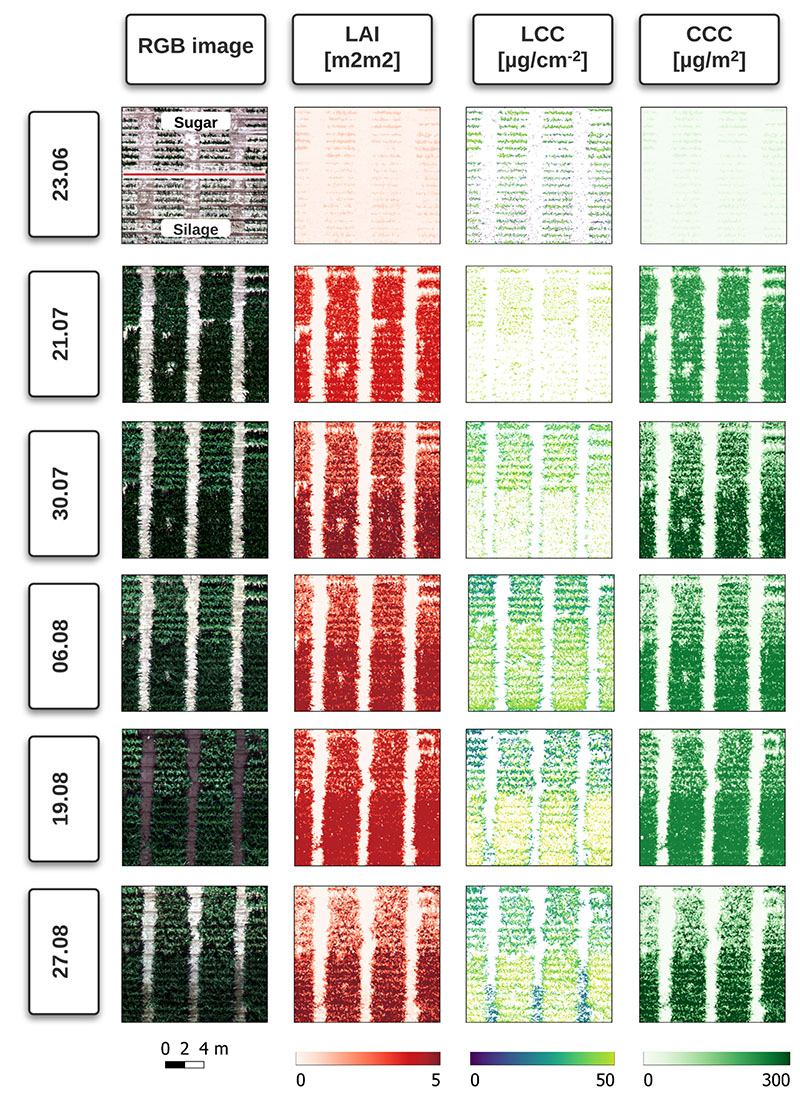
Maps of LAI, LCC and CCC on acquisition dates. RGB orthomosaics are depicted at 0.015 m spatial resolution. The first RGB map (23.06.) displays the separation between the two maize types. For fast processing, LAI and CCC maps were created using 0.09 m resolution orthomosaics. LCC maps are displayed at original resolution of 0.015 m.

**Figure 4:**
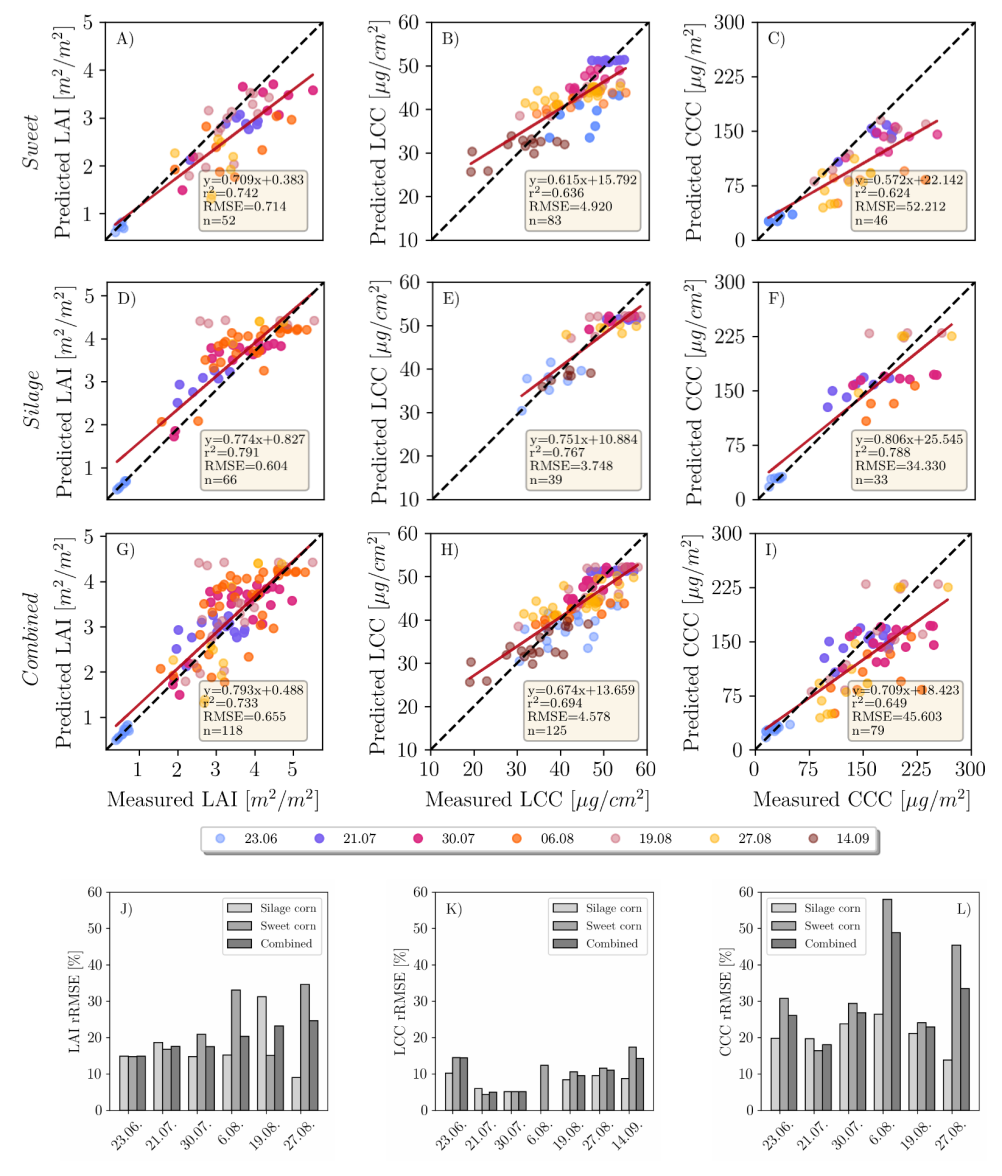
Comparison of predicted mean LAI, LCC and CCC values per subplot to the reference measurements throughout the growing season. The first row represents the inversion results for the mean reflectance approach applied to the sweet maize plots (**A**–**C**), second row—silage maize plots (**D**–**F**) and third row—both maize types (**G**–**I**). rRMSE plots for each date and variable are displayed in the lowermost row (**J**–**L**).

**Figure 5:**
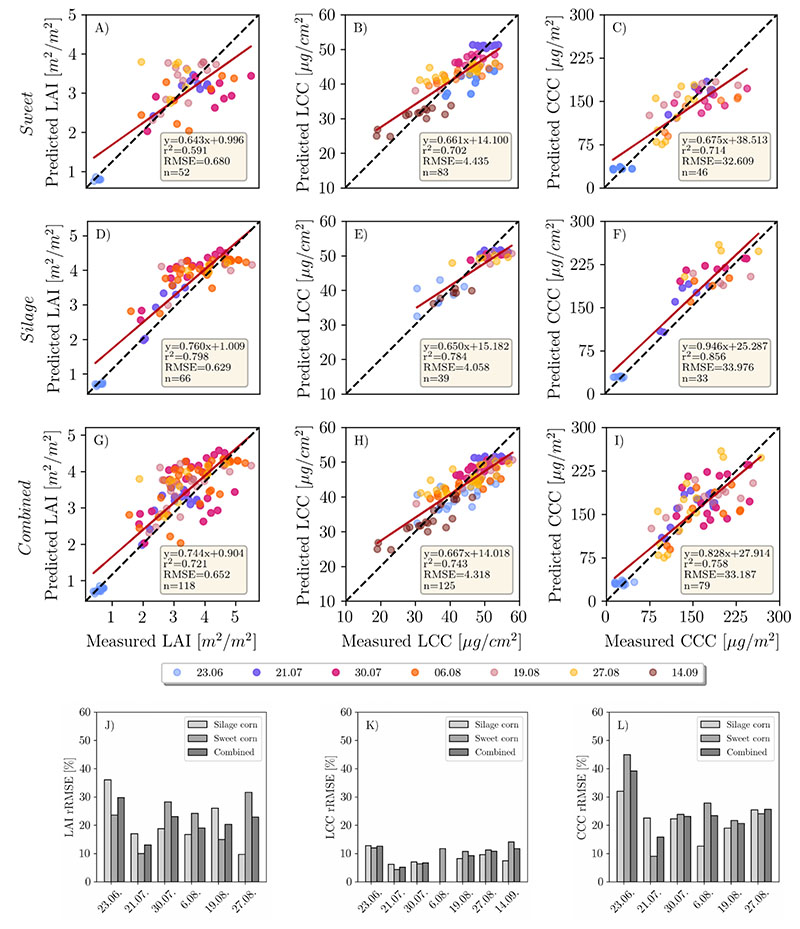
Comparison of predicted mean LAI, LCC and CCC values per subplot to the reference measurements throughout the growing season. The first row represents the inversion results for the pixel-based approach applied to the sweet maize plots (**A**–**C**), second row—silage maize plots (**D**–**F**) and third row—both maize types (**G**–**I**). rRMSE plots for each date and variable are displayed in the lowermost row (**J**–**L**).

A visual overview of the retrieval results for all dates can be found in Figure 3. Results of the LAI, LCC and CCC retrieval using the mean reflectance approach and pixel-based approach can be found in Figures 4 and 5, respectively. For LCC retrieval, mean reflectance values were calculated on pure green, sunlit pixels. For LAI and CCC retrieval, mean reflectances of soil, shadows and plant pixels were included. In the following sections, each variable will be analyzed by comparing the two above-mentioned approaches.

#### 3.1.1 LAI

**Figure 6:**
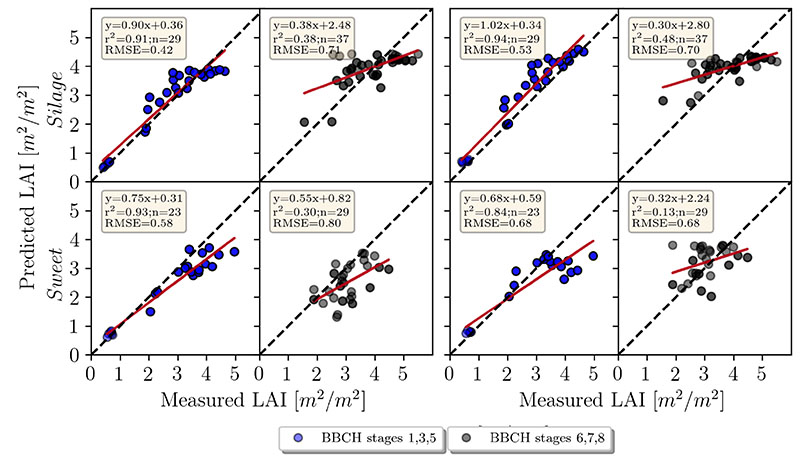
LAI retrieval using two approaches (**left** mean reflectance, **right** pixel-based) for early and late growth stages. BBCH principal growth stages 1, 3 and 5 correspond to leaf development, stem elongation and the start of inflorescence. Stages 6, 7 and 8 correspond to flowering, fruit development and ripening.

The accuracy of the LAI retrieval for silage maize is higher (RMSE = 0.604 m^2^/m^2^) compared to sweet maize (RMSE = 0.714 m^2^/m^2^) when looking at the pooled data of the mean reflectance approach (Panels A and D in Figure 4). There is a general trend of overestimation for the silage maize and underestimation for the sweet maize. When looking at separate dates (Figure 4J), the retrieval accuracies were higher in earlier plant growth stages (23.06., 21.07., 30.7.) compared to late stages. This is well depicted in Figure 6. Among these dates, the highest prediction accuracy could be achieved on 23.06., which corresponds to the growth stage of early stem elongation (rRMSE = 14.9%, Figure 4J), followed by late stem elongation (21.07., rRMSE = 17.6%) and start of heading (30.07., rRMSE = 17.5%). Retrieval accuracy decreased for sweet maize on 06.08. (flowering, rRMSE = 30%), while it remained stable for silage maize (rRMSE = 15%). The reason for this substantial decrease in the retrieval accuracy for the sweet maize might be attributed to the pollination, which covered plant leaves with a thin layer of yellow pollen, affecting the reflectance signal. On 19.08. (end of flowering, start of fruit development), we observed the opposite trends: low retrieval accuracy for silage maize (rRMSE = 31%) and high accuracy for sweet maize (rRMSE = 15.1%). This was unexpected, since, for the following measurement date (27.08.—fruit development), the trend again reversed (silage maize, rRMSE = 9%; sweet maize, rRMSE = 34%). One of the explanations for this shift might be the illumination conditions on 19.08. (cloudy) compared to other dates, when it was predominantly sunny. The underestimation of LAI in sweet maize might be attributed to the SunScan measurements, during which a fixed ELADP value was used. As mentioned before, sweet and silage maize had different LADs (leaf angle distribution). We used average ELADP for both maize types, as suggested in the manual. Thus, differences in LAD were not taken into account. Furthermore, LAD slightly changes over the plant growth period and this change was not reflected in the selected ELADP value.

In the pixel-based approach (Figure 5G), the total accuracy decreased only slightly (RMSE = 0.65 m^2^/m^2^) compared to the mean reflectance approach. Accuracy slightly increased for sweet maize (average rRMSE = 22%) and decreased for silage maize (average rRMSE = 20%) (Figure 5J). The highest rRMSE was determined for the silage maize dataset recorded on 23.06. (rRMSE = 36%), while sweet maize rRMSE also increased (23%) compared to the mean reflectance approach. In both cases, the models were overestimating.

#### 3.1.2 LCC

Compared to LAI, LCC retrieval produced more reliable results when using the mean reflectance approach. RMSE for silage maize (RMSE = 3.74 μg/cm^2^) was lower than for sweet maize (RMSE = 4.88 μg/cm^2^) (Panels B and E in Figure 4). It has to be noted that the numbers of samples taken in sweet maize plots were double the size compared to silage maize. Additionally, sweet maize in general was characterized by a larger variation in chlorophyll content at later growth stages, while LCC in silage maize within single dates showed little variation, except for 06.08., when the pollination was observed (Table 3). The coefficient of determination for pooled data is also higher for silage maize (r^2^ = 0.76) than for sweet maize (r^2^ = 0.63; Figure 4H). As for the single dates, the highest rRMSEs were recorded on 23.06. (early stem elongation, rRMSE = 14.5%) and 14.09. (start of senescence, rRMSE = 17.4%) for sweet maize (Figure 4K). The lowest rRMSEs were recorded for the datasets where LCC ground measurements had small Stdev (Table 3). The lowest chlorophyll content for sweet maize was retrieved for the dataset acquired on 14.09., when all sweet maize varieties were approaching senescence (Figure 4H). On the same date, the upper leaves of silage maize were still green, but decreased chlorophyll values were observed compared to 27.08. (Table 3). A general trend of overestimation is observed for low chlorophyll content, while higher LCC is characterized by underestimation (Figure 4H).

When looking at the pixel-based approach, the total estimation accuracy increased slightly (RMSE = 4.318 μg/cm^2^), driven by the accuracy increase in sweetmaize (Figure 5H). Here, again, we observe overestimation with low LCC values and underestimation with high values Figure 5H). For silagemaize, accuracy slightly decreased on 23.06. (rRMSE = 12%) and increased on 14.09. (rRMSE = 7.4%; Figure 5K).

#### 3.1.3 CCC

CCC retrieval is largely influenced by the LAI when looking at the mean reflectance approach (Figures 4I and 5I). The observed underestimation of LAI for sweet maize also caused an underestimation in the CCC (RMSE = 52.21 μg/m^2^, Figure 4C). When looking at single dates, the highest rRMSE (58%) was produced for the 06.08. (flowering) followed by 27.08. sweet maize datasets (rRMSE = 45.4%; Figure 4L). For silage maize, the achieved results were better (rRMSE across all dates ≈ 20%; Figure 4L). The retrieval accuracy drastically changed when applying the pixel-based approach (Figure 5I). A distinct decrease in RMSE from 52.21 to 33.18 μg/m^2^ (Figure 5C) could be observed for sweet maize. The main reason for the improvement was the distinctly reduced underestimation of predicted sweet maize LAI values (Figure 5I).

## 4 Discussion

In this study, the advantages of high-resolution imagery for maize crop variable retrieval using the PROSAIL model were demonstrated. The retrieval of LCC benefited from using a high spatial resolution to differentiate between distinct features in the scene, such as soil, shadow and plant pixels. For LAI, retrieval appeared to be challenging for the maize canopy. A clear discrepancy in LAI retrieval accuracy between different growing stages could be observed. We achieved an RMSE of 0.65 m^2^/m^2^ for the combined maize dataset retrieving LAI using the PROSAIL model (Figure 4G), which is comparable with the results observed for maize in satellite-based studies [17,18,53]. Comparison of our results to other UAV-based studies is challenging due the low number of studies focusing on high-resolution UAV-based variable retrieval with PROSAIL. Su et al. [54] reported an RMSE of 0.33 m^2^/m^2^ for a maize experiment with different sowing densities and across the entire growth period (which relates to our study) using a four-band multispectral camera. Our experiment, however, did not include different sowing densities, which tend to create significant contrasts in LAI. These contrasts are largely driven by the exposed soil, which differs by area based on the sowing density. In another study [38], the authors achieved an RMSE of 0.58 m^2^/m^2^ using hyperspectral UAV data of maize with a spatial resolution of 0.7 m. It has to be noted that the number of samples they used was very limited (four samples in total), and thus the comparison across growing stages cannot be made.

One of the reasons for the different LAI prediction accuracies achieved for sweet and for silage maize (RMSE = 0.714 and 0.628 m^2^/m^2^, respectively) might be the particular choice of the ELADP value used for leaf angle distribution during the SunScan measurements. In both maize types, a fixed value was used; however, the LAD visually differed between the two maize types. Therefore, the observed overestimation of LAI in the case of silage, and underestimation in the case of sweet maize, could be attributed to the fixed ELADP value chosen for the ground measurements. Additionally, the SunScan measurement results varied strongly depending on the probe placement in the canopy during data acquisition, which had a significant effect on the LAI estimation. Neighboring silage maize plots might also cast shadows on the sugar maize plots, distorting the measurements in the latter.

The discrepancy in LAI estimation at early (stem elongation) and late growth stages (flowering, etc.) is another point that requires attention. Better estimation results for early stages can be attributed to the smaller number of leaves compared to later growth stages, when leaf clumping is more pronounced, especially for nadir imagery, where row effects are clearly discernible [55,56]. It is known that PROSAIL simulations have limited applicability in row crops such as maize [57], which are characterized by large gaps between the rows and rather open canopies. Similar to [56], the underestimation observed specifically in sugar maize (Figure 4A) could be attributed to the absence of leaf clumping correction in the PROSAIL model. While these canopy characteristics were partially considered when conducting ground measurements, they cannot be parameterized in PROSAIL. PROSAIL assumes a homogeneous and closed canopy, the condition partially met by the satellite and airborne images but not by the high-resolution UAV images. For better estimation of LAI in maize, RTMs that account for leaf clumping and shading can be explored. Moreover, 3D radiative transfer models such as DART [20] consider the complex canopy structure and thus leaf clumping. However, for practical purposes, 3D RTMs have some disadvantages, such as their high computational demand and the larger number of parameters needed for model parameterization, which limits their application for agricultural practices.

Regarding the comparison of the pixel-based and mean reflectance approaches, we did not see significant improvements when using the pixel-based approach for LAI estimation. The latter generally overestimates the LAI values for both maize types compared to the mean reflectance approach (Figure 5). In the case of silage maize in the early growth stages, the measured RMSE for the mean reflectance approach was lower than for the pixel-based approach, yet underestimation was observed for high LAI values. This is not the case for the pixel-based approach, which is characterized by overestimation at all growth stages, resulting in a slope value of 1.02, as contrasted to a slope of 0.90 for the mean reflectance approach (Figure 6). The general overestimation of the pixel-based approach can be attributed to the usage of every pixel in the inversion. The shaded pixels, which cover larger areas in silage maize plots than the sunlit leaf pixels, result in higher LAI values compared to the sunlit green pixels (Figure 3). This in turn results in overestimation of LAI. The overestimation is apparent with the mean reflectance approach for the silage maize, where the shaded pixels distinctly contribute to the overall reflected radiation. The sugar maize is mostly characterized by a denser canopy, with shaded pixels only between the rows (see RGB images in Figure 3) and overall smaller areas covered by shaded pixels as compared to silage maize. The amont of LAI underestimation observed in sugar maize is in agreement with the findings of [56,58].

Although there is evidently a substantial benefit in using the very high-resolution imagery for LAI retrieval in breeding plots, for agricultural applications, it is not so beneficial. High-resolution imagery has proven advantageous for better discrimination between the pure plant pixels and the soil/shadows for biochemical parameter retrieval. We achieved high accuracy in LCC estimation by removing soil/shadows from the scene, when using the mean reflectance approach (combined RMSE 4.6 μg/cm^2^). Additionally, LCC retrieval benefited from the pixel-based approach (combined RMSE from 4.6 to 4.3 μg/cm^2^; combined r^2^ from 0.69 to 0.75). The results deliver better estimates than satellite-based studies (RMSE 8–10 μg/cm^2^) [56,58,59]. LCC estimation worked better for sweet than for silage maize. A possible explanation could be the low fractional cover in silage compared to sweet maize during the early growth stages—for example, on 23.06.—which led to limited vegetation pixels retrieved from the scene.

Furthermore, the in situ, non-destructive measurements may contribute to uncertainties in LCC retrieval. SPAD calibration equations are species- and variety-specific [60,61], but a fixed equation adopted from the literature was used in this study. Retrieval accuracy could be improved by deriving separate calibration equations per maize type, converting SPAD measurements to chlorophyll content in physical units. The constant overestimation of low LCC and underestimation of high LCC values by both approaches (Figures 4H and 5H) for each date may be attributed to the use of a calibration equation that does not reflect the actual relationship between SPAD values and real chlorophyll content.

We used all available spectral bands provided by the MicaSense Dual camera system for the LCC estimation. It is known that chlorophyll retrieval is mostly sensitive in the visible spectral range [51]. One of the ways to increase the LCC retrieval accuracy would be to apply a spectral constraint to the model. This would entail removing the spectral bands that are not sensitive to chlorophyll content from the reflectance data. As demonstrated previously [51], the removal of red-edge bands, characterized by the largest error between simulated and measured reflectance in maize, enabled higher retrieval accuracy.

Being a product of LAI and LCC, CCC retrieval accuracy depends on the retrieval of the two state variables. We found significant improvements in CCC retrieval when using the pixel-based approach (RMSEs reduced from 45.6 to 33.1 μg/cm^2^, and r^2^ from 0.64 to 0.75). This increase in accuracy is driven by the better estimation of LCC when applying the pixel-based approach.

Although the pixel-based approach delivers better results for LCC and CCC retrieval, the computing time of applying a LUT-based inversion scheme to each pixel is not practical. Compared to numerical optimization, the LUT-based approach is not as computationally intensive when the inversion scheme is applied to single plots, but it becomes inefficient when mapping the high-resolution orthomosaics of large areas. In this regard, hybrid approaches, which employ the power of machine learning combined with simulated spectra produced by an RTM, can distinctly speed up the process [10,62].

We demonstrated that high spatial resolution is beneficial for pigment retrieval, as well as LAI retrieval for small breeding plots, but we did not explore the impact of artificially reducing the spatial resolution on the final results. For application in large farming fields, UAVs need to be flown at much higher altitudes, producing orthomosaics with lower resolution. Thus, the effects of different spatial resolutions on the retrieval accuracy has to be better understood and studied in the future. Furthermore, a better understanding of the impact of LAD in both maize types is required. In this regard, plant architecture reconstructed from terrestrial laser scanners (TLS) [63] could potentially deliver information on leaf inclination angles that can be used to better parameterize the model and explore their impact on the parameterization.

## 5 Conclusions

In this study, we investigated the potential of the inversion of the radiative transfer model PROSAIL to retrieve LAI, LCC and CCC of sweet and silage maize, using spatial high-resolution UAV data acquired throughout one growing season. Two different retrieval approaches were investigated: (1) the mean reflectance approach—applying the inversion scheme to the mean reflectance spectrum per plot; (2) the pixel-based approach—applying the inversion scheme to all pixels of an orthomosaic and then calculating the variable mean for each plot. The performance of both approaches was evaluated based on the goodness of fit parameters RMSE and rRMSE by comparing the estimated variables to ground-truth measurements collected in the field.

Compared to spatially lower-resolution satellite and airborne imagery, high-resolution UAV images allowed the separation of soil, shaded and sunlit pixels. Thus, it was possible to retrieve the leaf chlorophyll content (LCC) by applying the inversion scheme only to green sunlit pixels. The LCC retrieval yielded promising results in comparison to ground measurements when using both retrieval approaches and led to higher accuracies compared to satellite or airborne studies. The measurement uncertainties associated with LCC retrieval could be further reduced by acquiring destructive chlorophyll measurements for a more accurate SPAD to LCC conversion equation. Furthermore, the impact of constraining the spectral range used for the retrieval of the different parameters should be further investigated. The retrieval of the structural variable leaf area index (LAI) was more challenging due to the mixing of sunlit and shaded pixels present in the UAV data. Further difficulties arose from plants grown in rows and having a complex canopy structure with varying leaf angles. The best results were obtained for early growth stages (leaf development, early and late stem elongation). We observed a significant improvement in the estimation of canopy chlorophyll content (CCC) when the pixel-based retrieval approach was used. We argue that high-resolution UAV imagery is well suited for biochemical variable retrieval, as shadows and background soil can be precisely removed, leaving only green plant pixels for the analysis. Compared to empirical approaches, vegetation RTMs offer a more robust, transferable solution to the retrieval problem and deliver results in real physical units. Further research is needed to validate the transferability of the model using similar sensor settings to the maize canopies. Furthermore, a more detailed characterisation of the canopy structure could improve the retrieval results, specifically of LAI. In addition, 3D RTMs would enable such a canopy characterization and should be explored in future studies.

## Supplementary Material

Appendix A

## Figures and Tables

**Table 1:** Centre wavelengths and bandwidths of the MicaSense Dual camera system.

Band Names	Centre Wavelength [nm]	Bandwidth [nm]
Blue444	444	28
Blue475	475	32
Green531	531	14
Green560	560	27
Red650	650	16
Red668	668	14
RE705	705	10
RE717	717	12
RE740	740	18
NIR840	842	57

**Table 2:** Details about flight campaigns in 2021. The scale of the Biologische Bundesanstalt, Bundessortenamt und CHemische Industrie (BBCH scale) was used to identify crop growth stages. SZA stands for solar zenith angle and was calculated for each date at 13:00 local time.

Date	Flight Time (CEST)	SZA	BBCH	Weather Conditions
**23 June**	13:08–13:25	27.81°	13–34	sunny
**14 July**	13:03–13:21	29.52°	39–55	partially sunny, few clouds
**21 July**	13:21–13:38	30.73°	-	sunny
**30 July**	13:20–13:37	32.71°	53–65	sunny, few clouds
**6 August**	13:19–13:40	34.55°	65–68	sunny, few clouds
**19 August**	13:35–13:53	38.55°	68–71	sunny, few clouds
**27 August**	13:17–13:33	41.33°	71–73	sunny, few clouds
**14 September**	13:27–13:40	48.14°	83–87	sunny, few clouds

**Table 3:** Statistics of leaf area index (LAI) and leaf chlorophyll content (LCC) field measurements collected for silage and sweet maize at CKA throughout the growing season; n—number of plots; Stdev—standard deviation, CV—coefficient of variation.

Variable	Maize Type	Stat	23.06	21.07	30.07	6.08	19.08	27.08	14.09
**LCC [μg/cm^2^]**	**Sweet**	**n**	11	11	11	11	6	20	13
		**Min**	39.0	46.9	41.8	34.2	30.3	31.6	19.0
		**Max**	53.1	54.3	53.2	54.7	53.5	53.4	42.6
		**Mean**	47.6	50.4	45.9	43.4	43.2	43.1	30.7
		**Stdev**	5.1	2.2	3.5	6.8	6.6	6.2	6.5
		**CV**	0.11	0.04	0.08	0.16	0.15	0.15	0.21
	**Silage**	**n**	7	6	7	-	7	6	6
		**Min**	30.6	48.9	46.1	35.5	41.9	40.8	35.3
		**Max**	44.2	56.9	56.5	56.8	57.9	61.4	53.6
		**Mean**	38.1	52.8	52.2	46.0	50.2	50.4	42.6
		**Stdev**	6.0	3.0	3.6	7.8	5.8	5.6	5.4
		**CV**	0.16	0.06	0.07	0.17	0.12	0.11	0.13
**LAI [m^2^/m^2^]**	**Sweet**	**n**	6	8	9	8	14	7	-
		**Min**	0.5	1.5	2.1	1.9	1.4	1.6	1.4
		**Max**	1.6	3.7	5.0	5.5	5.1	3.2	3.9
		**Mean**	0.9	2.9	3.5	3.5	2.9	2.6	2.9
		**Stdev**	0.3	0.8	0.8	1.0	0.8	0.5	0.6
		**CV**	0.37	0.27	0.22	0.30	0.28	0.19	0.22
	**Silage**	**n**	7	7	15	27	6	4	-
		**Min**	0.5	2.0	1.9	1.6	2.1	2.8	1.6
		**Max**	0.7	3.3	4.6	5.3	5.6	5.2	5.2
		**Mean**	0.5	2.5	3.5	3.9	4.1	3.9	3.6
		**Stdev**	0.1	0.5	0.9	0.9	1.2	0.9	1.3
		**CV**	0.18	0.21	0.24	0.24	0.30	0.23	0.35

**Table 4:** PROSAIL variables used in the construction of individual LUTs. LAI and LCC ranges were adjusted for each LUT separately.

Variable	Description	Range	Distribution
**PROSPECT-5**			
N	Leaf structure index	1.2–1.8 [26]	Uniform
C_ab_/LCC [μg/cm^2^]	Leaf chlorophyll content	0–70	Gaussian
C_cx_ [μg/cm^2^]	Leaf carotenoid content	Default value	-
C_bp_ [unitless]	Brown pigments	0–0.5	Fixed/Uniform
C_m_ [g/cm^2^]	Dry matter content	0.004–0.0075 [26]	Uniform
C_w_ [g/cm^2^]	Leaf water content	Default	-
**4SAIL**			
LAI [m^2^/m^2^]	Leaf area index	0–7	Uniform
ALIA [°]	Average leaf inclination angle	20–70 [26]	Step of 1
Hot	Hot spot parameter	0.01–0.5 [26]	Uniform
*ρ*_soil_ [%]	Soil reflectance	Extracted from image	-
SZA [°]	Sun zenith angle	Different for each date	-
OZA [°]	Observer zenith angle	0	-
rAA [°]	Relative azimuth angle	0	-

## Data Availability

Not applicable.
