# Supplementary material for: Retrieval of Crop Variables from Proximal Multispectral UAV Image Data Using PROSAIL in Maize Canopy
Source: Remote Sens (Basel). Author manuscript; Available in PMC 2022 Sep 7. (PMC7613386; doi:10.3390/rs14051247)
Supplement: Appendix A [file EMS152680-supplement-Appendix_A.pdf]

Appendix A

Appendix A.1

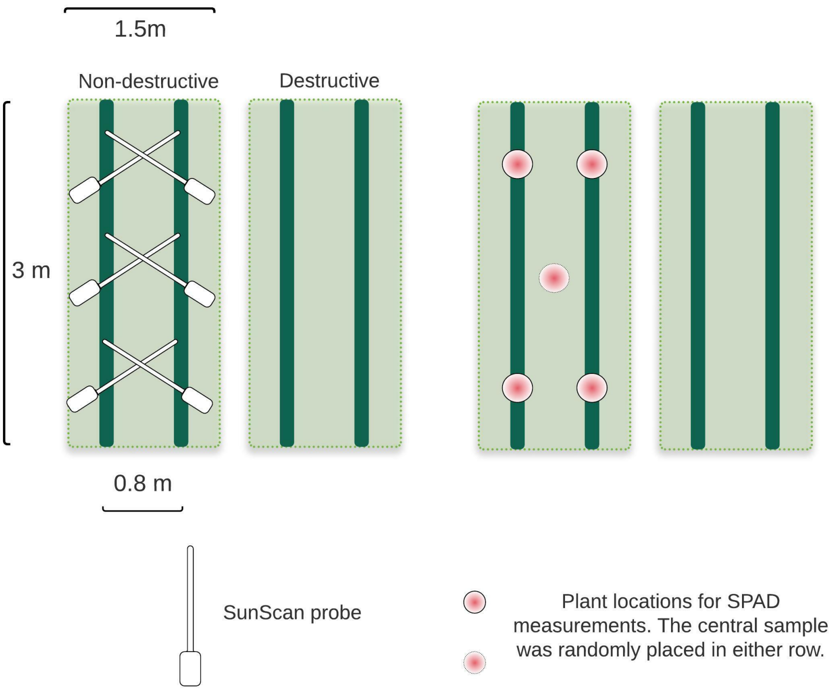

Figure A1. SunScan probe placement (**left**) and SPAD measurement locations (**right**). Thick green lines represent maize rows.

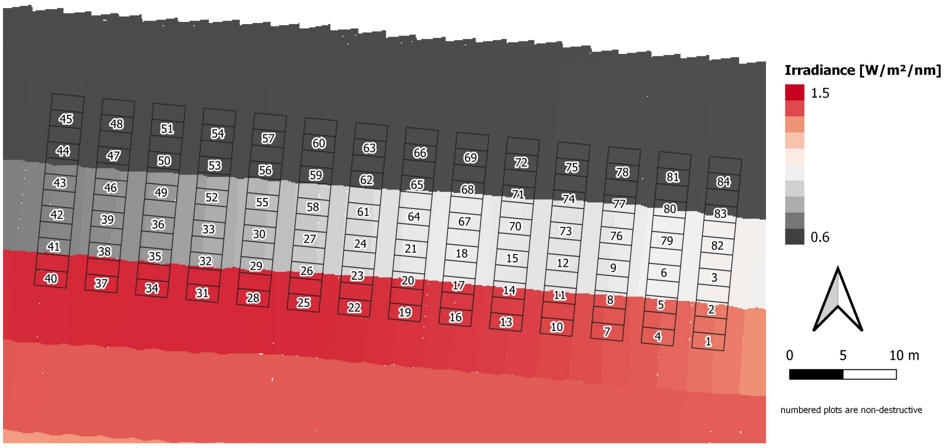

Figure A2. Map of the irradiance measurements taken by the DLS for each image on 14 July 21. These measurements are depicted for one band only.
